# Video Review

**DOI:** 10.1120/jacmp.v3i2.2583

**Published:** 2002-03-01

**Authors:** Peter R. Almond

**Affiliations:** ^1^ Department of Radiation Physics‐94 M. D. Anderson Cancer Center University of Texas 1515 Holcombe Blvd. Houston TX 77030

## Abstract

**Is radiation as dangerous as they say?** John Cameron, Medical Physics Publishing, Madison, WI 2002.

PACS number(s): 87.50.Gi, 87.52.Px, 87.52.Tr

Although this is not a book, this video is sold by Medical Physics Publishing and appears in their 2002 Spring‐Summer Catalog. It is a video lecture that was prepared for a Medical Effects of Ionizing Radiation (MEIR) Course at the Armed Forces Radiobiology Research Institute (AFRRI) in August 2001. Along with the video there is a 12‐page supplement. This consists of a hard copy of the slides shown in the video, a three‐page article by Cameron, “Is radiation an essential trace energy?” and a short abstract, “Promoting Understanding of Radiation in the Radiology Clinic,” both unpublished, but each with a short reference list. These articles cover most of the material in the video.

It would have been helpful to know the subject matter of the other lectures and the context in which they were presented. Cameron's thesis is that a little radiation rather than being harmful is, in fact, of benefit, especially to older people. This is opposite to contemporary wisdom so that this video needs to be shown at the same time that that the opposite view point is given and with a moderator to facilitate discussion of questions raised by the contrast between the two. One can only hope that this was done when the video was used for its original purpose. Unfortunately the Medical Physics Publishing catalog seems to promise more than was contained in the video. The catalog says, “… he (Cameron) challenges the generally accepted linear no threshold (LNT) hypothesis and presents the results of several studies to back his argument.” However, the linear no threshold theory is not mentioned or alluded to once in the video, nor as far as I could see, in the supplementary material. This is not a balanced presentation giving the strengths and weakness of both viewpoints.

This is the presentation of an evangelist preaching his gospel of safe radiation. Certainly Cameron has the right to his viewpoint and the right to present it and indeed there might be some validity to what he says, but there are too many flaws in this presentation to come to any other conclusion than to say that it is an interesting idea.

Cameron presents the results of several studies to back up his argument. Only one of these studies has been published in a peer reviewed scientific publication. That is the study of the mortality of British radiologist from 1900 to 1980 by Smith and Doll published in the British Journal of Radiology in 1981. This data is interesting and perhaps it is surprising that no one, as far as I know, has seriously followed this up. It does on the surface seem to support Cameron's viewpoint. So does the study of nuclear shipyard workers in which Cameron participated. Again this is a long‐term study of workers exposed to radiation versus those who were not but it has never been published in a peer reviewed scientific journal, although the report is apparently available from the Department of Energy (Matanoski *et al*., DOE DE‐AC02–79 EV10095).

Unfortunately Cameron makes much in his lecture on the variation of natural background radiation in the U.S. and cancer rates. He shows a slide of the background radiation in the U.S: Lower in the coastal Gulf and East coast regions and higher in the middle part of the country. He then shows the cancer mortality rates across the U.S: Higher in the coastal Gulf and East coast regions and lower in the middle part of the country. Cameron would have us believe that this proves his case. He says nothing about the fact that much of the county's oil refineries, chemical and industrial plants are along the Gulf coast and eastern seaboard. These might be far more significant factors in the cancer morality rates than his hypothesis that the higher levels of background radiation in the middle part of the country has stimulated that population's immune system and protected them from getting cancer.

He makes the same kind of unfounded and flawed arguments concerning radon and lung cancer in the U.S. This is unfortunate because there is a radiation phobia in this country. This has proved to be very expensive in terms of meeting the very low and to many, including Cameron, unnecessarily low, regulatory radiation levels. This has also clearly hurt the country in its attitude toward nuclear power. All of this Cameron points out and as such he has a case to make, but much of it is negated by his highly selective presentation of the data.

He has suggested that to combat radiation phobia, patients undergoing procedures with radiation have their radiation dose explained in terms of “Background Equivalent Radiation Time,” otherwise known as BERT. That is, you would tell a patient getting a chest x‐ray that the amount of radiation they are getting is equivalent to seven days of natural background radiation. Most patients I think would buy that. However, if they get a whole body CT scan they would use up four years of background radiation. I'm not sure how many patients would like that!

There is more in this video. Cameron would like to promote a double blind clinical trial to prove his hypothesis. People in retirement homes would have boxes under their beds. Some boxes would contain crushed granite and so increase their radiation background dose, while others would have ordinary crushed rocks and no increased dose. They would be followed to see if the higher radiation dose group lived longer with less illness because their immune system had been stimulated by the radiation as compared to the low radiation group. As Cameron correctly points out, he will not see this study done in his lifetime (although he did volunteer to be the first participant in it), nor I expect will it be done in the lifetime of most of the readers of this journal.

I found the lecture entertaining and interesting, although I wished that the audio had been a little clearer. Also it was not possible to see the details in some of the slides both on the screen and in the handout. Although the video cannot be changed it should be possible to make sure that the copies of the slides in the handout are sharp and readable.

Having pointed out some of the flaws in Cameron's presentation I still think that this video has a place in teaching institutions. It could be used in any lecture series dealing with radiation protection, regulatory issues, radiation effects, radiobiology, etc., but only as part of a series of lectures that presents all sides of the issue and hopefully with an instructor that can bring a balanced approach to this subject. It could also be used as a starting point in a discussion with the students on the issues raised by this review of the video presentation itself.

